# SnowMotion: A Wearable Sensor-Based Mobile Platform for Alpine Skiing Technique Assistance

**DOI:** 10.3390/s24123975

**Published:** 2024-06-19

**Authors:** Weidi Tang, Xiang Suo, Xi Wang, Bo Shan, Lu Li, Yu Liu

**Affiliations:** 1 Key Laboratory of Exercise and Health Sciences of Ministry of Education, Shanghai University of Sport, Shanghai 200438, China; weidi_tang@sus.edu.cn (W.T.); zwx252@163.com (X.W.); 2321518059@sus.edu.cn (B.S.); lilu@sus.edu.cn (L.L.); 2 School of Athletic Performance, Shanghai University of Sport, Shanghai 200438, China; xiang_suo@sus.edu.cn

**Keywords:** wearable sensors, motion capture, digital human, mobile application, data visualization

## Abstract

Skiing technique and performance improvements are crucial for athletes and enthusiasts alike. This study presents SnowMotion, a digital human motion training assistance platform that addresses the key challenges of reliability, real-time analysis, usability, and cost in current motion monitoring techniques for skiing. SnowMotion utilizes wearable sensors fixed at five key positions on the skier’s body to achieve high-precision kinematic data monitoring. The monitored data are processed and analyzed in real time through the SnowMotion app, generating a panoramic digital human image and reproducing the skiing motion. Validation tests demonstrated high motion capture accuracy (cc > 0.95) and reliability compared to the Vicon system, with a mean error of 5.033 and a root-mean-square error of less than 12.50 for typical skiing movements. SnowMotion provides new ideas for technical advancement and training innovation in alpine skiing, enabling coaches and athletes to analyze movement details, identify deficiencies, and develop targeted training plans. The system is expected to contribute to popularization, training, and competition in alpine skiing, injecting new vitality into this challenging sport.

## 1. Introduction

With the rapid advancements in integrated circuits and artificial intelligence technologies, data acquisition and analysis methods in the sports industry are undergoing an unprecedented transformation. Massive amounts of training and event statistics data provide researchers with a more multidimensional model of the real world, opening up grand perspectives that traditional manual analysis methods can hardly achieve. By analyzing and organizing big data, researchers can explore new mysteries of sports from unprecedented angles and deconstruct and explore the meaning of data in entirely new ways [1]. In the field of alpine skiing, which combines speed and intricacy, researchers are investigating the quantitative impact of factors such as center of mass, air resistance, and skiing trajectories on athletes’ performance [2,3,4]. By relying on motion capture technology, researchers can conduct in-depth kinematic analyses of skiing technique movements, tailoring targeted training and competition plans for each athlete. Abundant real-time motion capture data help identify inefficiencies, optimize movement patterns, and develop training plans tailored to individual skiers’ needs. The existing literature highlights the importance of joint angles as key biomechanical factors influencing the technique and performance of elite alpine skiers [5,6,7]. The findings emphasize the crucial role of these joint angles in force generation, dynamic balance, and skiing technique, providing valuable insights for assessing and optimizing the performance of high-level skiers [8]. However, existing skiing monitoring systems still have many deficiencies in kinematic data monitoring: most applications rely on integrated sensors in smartphones or other portable devices, resulting in low motion capture accuracy or reliability [9,10]. Furthermore, corresponding data processing and analysis algorithms need further improvement; for example, the motion assessments and recommendations provided by products like CARV and SNOWCOOKIE require refinement [11,12].

In this study, the developed system, SnowMotion, is a digital human motion training assistance platform for skiing based on wearable sensors. The system utilizes wearable sensors to acquire the user’s trunk and lower limb kinematic information, capturing and analyzing the skier’s movements in real time. It achieves a new balance between system accuracy and usage complexity, providing users with accurate and instructive skiing assistance information through reliable data acquisition and analysis, combined with visualization interaction design tailored for skiing scenarios. We hope to provide skiers, coaches, and enthusiasts with a novel training assistance tool through this system. By precisely capturing and analyzing skiing movements, the system can identify key factors affecting performance and provide users with targeted improvement suggestions. This not only helps enhance competitive levels but also reduces the risk of sports injuries, promoting the safe and healthy development of skiing.

## 2. Methods

### 2.1. Related Works

Wearable sensor-based motion capture is a technology that utilizes sensors (such as accelerometers, gyroscopes, and magnetometers) attached to the human body or objects to measure the movement and orientation of various parts. Compared to other methods, this technology has advantages such as portability, flexibility, and a low cost. However, it also has some specific drawbacks, such as difficulties in sensor calibration, complex data fusion processing, drift correction, and noise interference. Cal-Net (Calibration Network) optimizes classification and calibration performances, eliminating the need for post-processing confidence calibration [13]. Fair-Net aims to reduce performance disparity across identifiable sub-populations, such as genders or racial groups [14]. These architectures could be applied to ensure accurate motion reconstruction across different user morphologies and demographics. By incorporating principles from Cal-Net, the system could adjust predictions for each user’s body schema, while a Fair-Net-like approach could enhance fairness and accuracy across different genders, body types, and morphological features.

One potential improvement for wearable sensor-based motion capture systems is the integration of self-powered sensors. Self-powered sensors in wearable devices offer innovative solutions for health monitoring and motion analysis. Ultra-flexible TENGs manufactured using 3D printing technology can power electronic devices and adapt to various shapes [15]. CF-TENG application in intelligent sports monitoring achieves the precise recognition of athletes’ movements through machine learning algorithms [16]. TENGs’ applications in healthcare, including microbial disinfection, interventional therapy, and implantable microsystems, emphasize the importance of energy harvesting, storage, and energy-efficient design [17]. However, energy supply is not the limiting factor for SnowMotion’s functionality and performance. While self-powered sensors offer continuous energy harvesting, they have higher manufacturing costs and more complex fabrication processes compared to battery-powered sensors. The energy consumption of battery-powered sensors is optimized through efficient algorithms and power management techniques.

In contrast to using wearable sensors, smartphone-based motion capture is more convenient and user-friendly. This technology does not require additional wearable devices and directly utilizes the built-in sensors of smartphones to achieve activity recognition or motion capture in specific scenarios. Behrooz et al. successfully implemented activity detection for alpine skiers using the inertial measurement unit (IMU) built into smartphones, achieving a 99.25% accuracy in motion recognition, demonstrating the accuracy and convenience of motion recognition based on smartphone IMU sensors [18]. However, due to the significant differences between the usage scenario design of smartphones and alpine skiing, the information obtained from smartphone built-in sensors is often less reliable [19]. In extreme scenarios such as alpine skiing, a more reliable choice is to use wearable sensors as the data acquisition source. Martínez et al. used pressure sensors to extract users’ skiing trajectories in turns and achieved precise digital modeling [20]. This method has been tested for its effectiveness in turns with different slopes and curvatures, showing considerable room for development. Building on this foundation, Snyder et al. introduced a scoring algorithm for evaluating the quality of alpine skiing movements. This algorithm utilizes an inertial measurement unit attached to ski boots and applies principal component analysis to identify sources of variation in the collected data, distinguishing skiers of different skill levels based on technical differences [21].

### 2.2. System Overview

**Figure 1:**
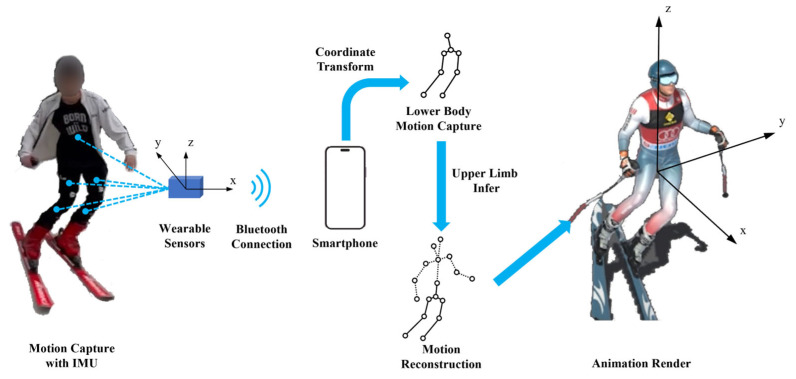
System overview. The sensors establish a Bluetooth connection with a smartphone. The smartphone performs the necessary coordinate transformations and motion reconstruction algorithms to generate a digital animation of the skier’s movements in real time.

As shown in Figure 1, the SnowMotion system employs five inertial measurement units for real-time, high-precision motion capture of the user’s lower limbs and trunk. These sensors are fixed to a ski suit and incorporate devices such as accelerometers, gyroscopes, and magnetometers to measure the kinematic parameters of the sensors. Accelerometers detect changes in vertical and horizontal acceleration, while gyroscopes measure angular velocity and rotation, providing information on the body posture and spatial position during skiing. Magnetometers detect the strength and direction of the magnetic field, aiding in measuring the skier’s orientation and position. The GPS determines the skier’s location and speed through satellite signals, enabling comprehensive monitoring and tracking of the skiing process. The mobile application platform consists of three main modules: sensor communication, data processing, and user interaction. The sensor communication module is responsible for sensor scanning, connection, and the configuration of wearing positions. The data processing module integrates, classifies, filters, and analyzes real-time kinematic data, including posture, speed, acceleration, and skiing distance, and utilizes 3D motion reconstruction for visualization. The user interaction module includes functions such as motion playback, information editing and storage, and display information adjustments.

Motion capture is performed by sensors fixed on the user’s lower limbs, and the acquired information is transmitted via Bluetooth from the wearable sensors to the smartphone platform app. The data undergo spatial coordinate transformation, lower limb motion capture, upper limb motion inference, and other processing steps. Finally, the smartphone’s graphic rendering function is invoked to reconstruct the digital human model, achieving real-time motion capture and three-dimensional reconstruction of skiing movements in the field.

### 2.3. Definitions of Coordinate Systems

When using wearable sensors for motion capture in actual application scenarios, the first step is to perform a reference coordinate system transformation from the sensor coordinate system (SCS) to the global coordinate system (GCS). The GCS provides a standardized and unified framework for locating and measuring objects on a larger scale, usually associated with geographic coordinate systems, allowing us to specify the position of any point on the Earth’s surface [22]. The GCS adopted in this study is a right-handed Cartesian coordinate system, with the X-axis pointing east, the Y-axis pointing north, and the Z-axis pointing upward.

This study used quaternions to complete the transformation from the SCS to the GCS. First, the sensor outputs a quaternion representing its current orientation, which describes the rotational relationship between the sensor coordinate system and the global coordinate system. Second, the orientation quaternion is normalized by dividing each component of the quaternion by its modulus to ensure that the modulus of the quaternion is 1. The normalized orientation quaternion is then multiplied with the SCS quaternion, which represents the coordinates of a vector or point in the sensor coordinate system. By multiplying the orientation quaternion with the SCS quaternion, we can transform the vector or point from the SCS to the GCS. This multiplication operation effectively rotates the SCS quaternion around the rotational axis represented by the orientation quaternion, aligning it with the GCS. Finally, the transformed vector is extracted from the result of the multiplication. The result is a new quaternion containing the transformed vector information. We can extract the transformed vector, i.e., the coordinate representation of the vector in the GCS, by taking the imaginary part of this quaternion.

### 2.4. Initial Attitude Calibration

Determining the initial pose reference of a human body in three-dimensional inertial human motion capture is crucial. This process is referred to as initial attitude calibration in motion capture. The essence of initial pose calibration is to find the transformation matrices from the SCS to the GCS for each keypoint. Euler angles use three angles to describe the rotation around three axes and are more commonly used in biomechanics, but they can cause gimbal lock and discontinuities. Quaternion angles use four numbers to describe the rotation around one axis, but they are less intuitive and harder to calculate. Given the calibrated pose, the transformation matrix between the SCS and the GCS can be computed using the output values of the accelerometer and magnetometer [23]. The computation formula is as follows:

(1) $$\varphi_{roll\;}=\arctan\left(a_{x},\;a_{z}\right)$$

(2) $$\theta_{pitch}=-\arcsin\left(a_{x},\;g\right)$$

(3) $$\psi_{yaw\;}=atan(m_{x}cos\theta +m_{y}sin\theta \;sin\varphi +m_{z}sin\theta \;cos\varphi ,m_{y}cos\varphi -m_{z}sin\varphi )$$

where the angle of rotation around the Z-axis is referred to as yaw, the angle of rotation around the X-axis is referred to as pitch, and the angle of rotation around the Y-axis is referred to as roll. The components of the accelerometer on the three axes are denoted by $a_{x}$, $a_{y}$, and $a_{z}$. Additionally, g represents the local gravitational acceleration. Similarly, the magnetometer provides components $m_{x}$, $m_{y}$, and $m_{z}$ along the three axes. In this system, the Z-Y-X rotation sequence is employed, allowing the representation of these three rotations using quaternions:

(4) qx=[cos⁡φroll 2,sin⁡φroll 2,0,0]

(5) qy=[cos⁡θpitch 2,0,sin⁡θpitch 2,0]

(6) qz=[cos⁡ψyaw 2,0,0,sin⁡ψyaw 2]

(7) $$q=q_{z}⊗q_{y}⊗q_{x}$$

The four components of quaternion q are defined as follows:

(8) q1=cos⁡φroll 2cos⁡θpitch 2cos⁡ψyaw 2+sin⁡φroll 2sin⁡θpitch 2sin⁡ψyaw 2

(9) q2=sin⁡φroll 2cos⁡θpitch 2cos⁡ψyaw 2−cos⁡φroll 2sin⁡θpitch 2sin⁡ψyaw 2

(10) q3=cos⁡φroll 2sin⁡θpitch 2cos⁡ψyaw 2+sin⁡φroll 2cos⁡θpitch 2sin⁡ψyaw 2

(11) q4=cos⁡φroll 2cos⁡θpitch 2sin⁡ψyaw 2−sin⁡φroll 2sin⁡θpitch 2cos⁡ψyaw 2

Due to the nature of the motion capture data collected in this system, which only include information about the lower limbs and torso, during the initial attitude calibration, strict requirements for the angles of the upper limbs in the user’s T-pose are no longer necessary. Only the lower limb posture undergoes fixed calibration. The user is instructed to face the x-axis direction and assume a T-pose for a few seconds to complete the initial attitude calibration.

### 2.5. Motion Reconstruction

This system is a specialized application developed for the mobile app usage scenario. Considering the performance limitations and practical situation of smartphones, it directly invokes the animation module of Unity to reconstruct and render the motion of the digital human, achieving the 3D motion reproduction function on the mobile end with low cost. The high-precision motion data are saved and compressed in real time during the skiing process and support a millimeter-level 3D motion reproduction function after being exported to a high-performance computer, which is convenient for athletes or coaches to watch the captured skiing motion information from any angle.

**Figure 2:**
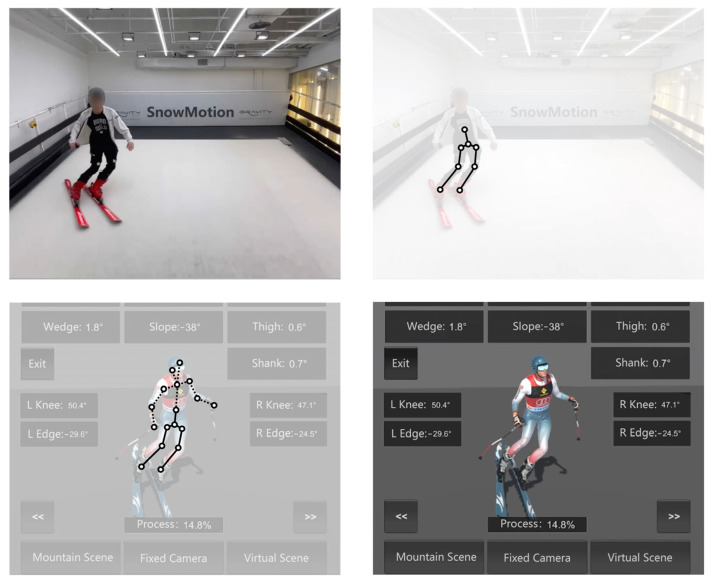
Motion reconstruction. Solid lines represent captured pose information, while dashed lines indicate inferred upper body and head motion. The top-left image depicts the real skiing scene, and the bottom-right image shows the reconstructed and rendered motion on a mobile device.

We create and control a human model in a virtual skiing environment. First, we define a class for each bone in the model, along with a method to rotate the bone relative to its parent. Then, we read the sensor data for the model and update the model’s motion in every frame based on the quaternion values. Our data collection process only captures the angles of the user’s lower limbs and torso. Hence, to achieve a more natural and realistic motion of the entire human body model, we infer the upper body movements based on kinematic models, which leverage the known relationships between body segments and the principles of biomechanics to estimate motion. In Figure 2, the solid line segments represent the actual captured motion pose information, while the dashed line segments represent the inferred upper limb and head motion information computed based on the actual pose information. The top left image depicts the real sliding scene, while the bottom right image illustrates the reconstructed and rendered motion scene on the mobile device. The core concept revolves around calculating the rotation angles of the upper limbs, taking into consideration the rotation angles of the lower limbs and the degree of inclination of the spine.

The system utilizes Unity’s animation module and defines classes for each bone to rotate relative to its parent. It reads sensor data and updates the model’s motion every frame based on quaternion values. For body parts not directly captured, like the upper limbs and head, it employs kinematic models and biomechanical principles to estimate motion. The architecture involves data collection, compression, animation module integration, human model definition, sensor data processing, kinematic inference, and mobile rendering components. The hyperparameters include the compression ratio, interpolation factors for kinematic inference, sensitivity of the kinematic model to lower limb angles, and performance optimization parameters for mobile devices. Adjusting these parameters would involve an iterative process, testing different values, observing the impact on motion reproduction accuracy and performance, and fine-tuning accordingly through development environments, user testing, and performance profiling to balance realism and mobile device capabilities.

### 2.6. User Interface and Visualization

**Figure 3:**
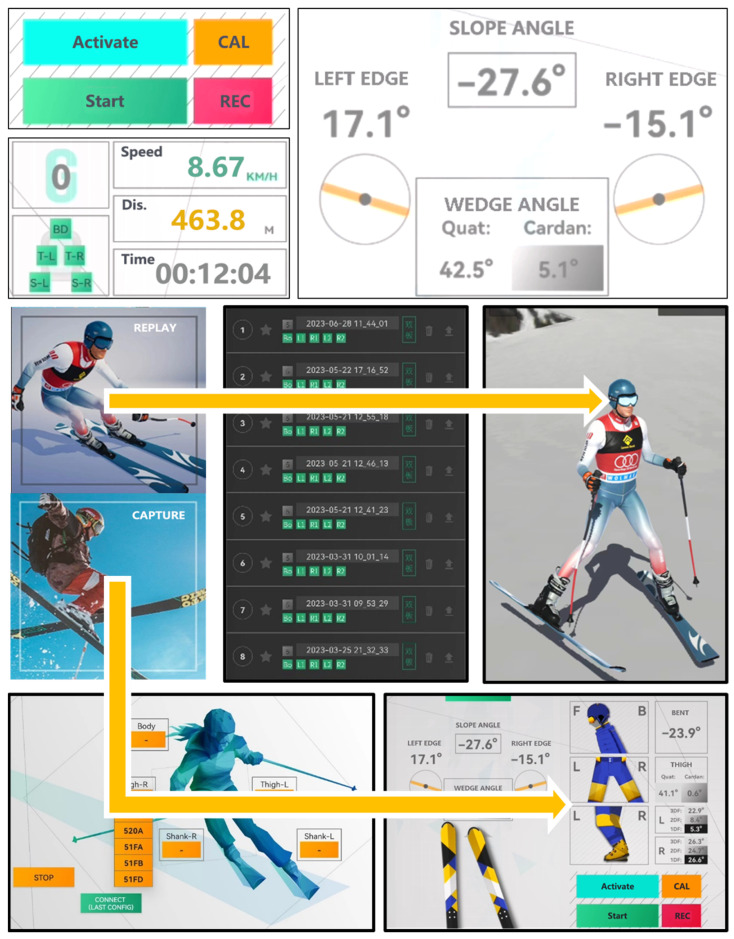
Visibility design and control flow. The top image shows the dashboard, while the middle-left image displays the first screen after launching the system. Users can access the two main features, skiing motion monitoring and 3D motion replay, with just two clicks.

The user interface influences the user’s interaction with and perception of an application. It should provide clear, intuitive, and engaging information and feedback to the user while minimizing cognitive load and distractions. Considering the unique characteristics of the application’s usage scenario, such as outdoor environments and low temperatures that may require users to wear gloves, touch interactions face challenges such as reduced accuracy, unreliable touch detection, and decreased screen visibility due to sunlight reflecting on the snow. To meet these specific requirements, as shown in Figure 3, we focus on improving the user interface design from two aspects:

Visibility: We utilized high-contrast colors, large fonts, and clear icons to ensure that user interface elements are visible and readable under bright and snowy conditions. Based on the level of importance, we used light yellow to represent buttons that are used only once and are of lower importance. We employed eye-catching colors to mark buttons that are needed for each use. The start button for motion capture is represented in low-pressure green. In case of emergencies, the emergency button for reconnecting sensors is highlighted in red. In the data display area, different colors are used to represent different types of data, enabling users to quickly obtain the desired data without carefully distinguishing the presentation location of the target data once they are familiar with the software interface. For the carving angle, which is crucial to the user, the only colored information in the entire functional area is the edge angle, providing users with intuitive monitoring of the angle. Precise motion information is then provided through the associated numerical data.

Simplicity: We minimized the number of user interface elements and options, prioritizing the most important and frequently used functions. In the initial interface of the software, we designed only two buttons: “Motion Capture” and “Motion Playback”. Users can quickly access the desired functionality with just two clicks, whether it is to perform motion capture or view existing records. We strived to reduce the number of elements in the application interface to a minimum, simplifying it as much as possible. This approach aims to avoid imposing additional usage costs on users by avoiding complex functional buttons.

## 3. Results

Through a series of targeted experiments and analyses, the accuracy and robustness of the SnowMotion system were thoroughly validated. The system’s sensor drift suppression capability under different conditions was tested through forced pendulum experiments, and the accuracy of the system in human motion capture tasks was verified by comparing it with the Vicon motion capture system under laboratory conditions.

### 3.1. Forced Pendulum Experiment

When using IMU sensors for motion capture tasks, the impact and error caused by drift cannot be ignored. Sensor drift refers to the deviation between the estimated orientation and position based on the sensor output data and the actual situation over time. These deviations originate from the accumulation of various disturbance factors, including but not limited to signal noise, instability, temperature changes, cross-axis sensitivity, and external interference [24]. Sensor drift can affect the accuracy and reliability of IMU data, directly impacting the performance of the motion capture system. This study employed forced pendulum experiments to examine the sensor drift suppression under different conditions and scenarios, evaluating the effectiveness of our data processing algorithms in suppressing drift errors [25].

**Figure 4:**
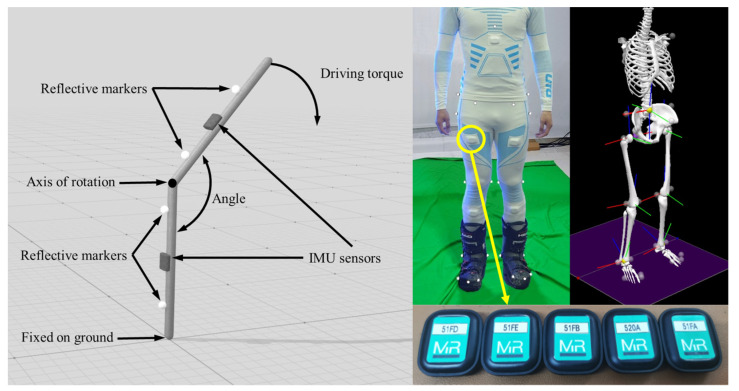
Experiment scene. (**Left**) Forced pendulum experiment to test system repeatability and sensor drift suppression using a fixed linkage structure on a horizontal plane. (**Right**) Verification of dynamic accuracy, where subjects wore sensors and performed specific movements for data collection and processing. The tri-color coordinate axes shown in the figure represent the local coordinate systems for each joint, with the blue axis denoting the X-axis, the red axis denoting the Y-axis, and the green axis denoting the Z-axis.

**Figure 5:**
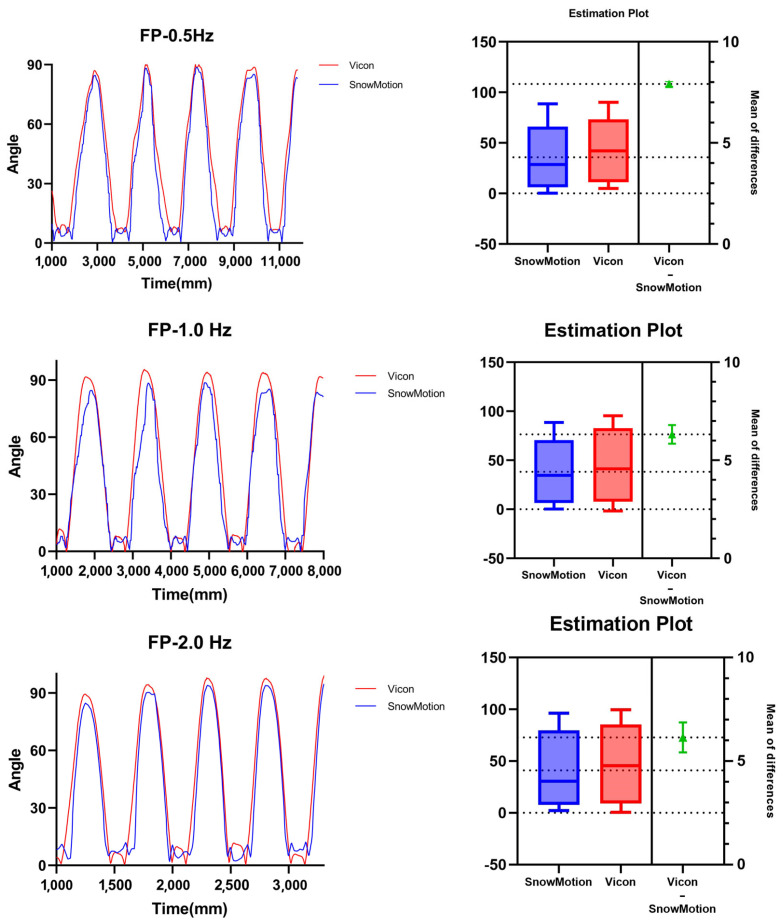
Waveforms at 0.5 Hz, 1 Hz, and 2 Hz. The three curves show four test cycles in each group; the statistical data were calculated based on the full-cycle test data.

To verify the effect of the data processing system on suppressing IMU sensor drift, we used the Vicon motion capture system to record the pendulum of two rod-shaped rigid bodies around the same fixed rotational axis. As shown in Figure 4(left), we attached our self-developed IMU wearable sensors to the two rod-shaped rigid bodies. According to relevant studies, one segment was fixed to the ground, while the rotation amplitude of the other segment was set to 40° to 90° [26]. Considering the fluctuation characteristics of the input signal in actual usage scenarios, we applied irregular periodic driving torques. We divided the frequency into three groups, 0.5 Hz, 1.0 Hz, and 2.0 Hz, with each group of motion tests lasting 30 s in total. We compared the IMU sensor data with the Vicon data to assess the accuracy and stability of the IMU sensors and the data processing system. The forced pendulum experiment curves are shown in Figure 5.

In the forced pendulum experiment, we compared the angle measurement accuracy of the SnowMotion system and the Vicon motion capture system. It can be observed that the proposed method in this study demonstrated good angle tracking accuracy and stability while achieving relatively stable performance in suppressing sensor drift. Table 1 lists the mean values, standard deviations, and correlation coefficients measured in the experiment for different frequency groups.

### 3.2. Verification of Dynamic Accuracy

It is generally believed that alpine skiing consists of three basic types of movements: straight gliding, gliding wedge, and carving. The combination and variation of these three movements form more advanced snow techniques [27,28,29]. During straight gliding, the two skis are in a parallel state, which is the most basic gliding movement. It can affect the pressure distribution of the skis and the overall center of gravity through the angles of the knees and thighs. Therefore, the relative angles of the knees and thighs to some extent reflect the skier’s skill and movement level [30]. The gliding wedge is a snow technique that controls the skis to form a wedge angle for braking and deceleration. The larger the angle between the skis, the greater the resistance obtained, but it is also more likely to cause accidents such as loss of control and falls; the smaller the angle, the more conducive it is to increasing the gliding speed, but the corresponding speed and control pressure also increase. In actual gliding, adjustments need to be made according to the snow conditions and subjective speed requirements [31]. Carving is considered a more complex technique. In addition to controlling the knee angle and hip joint angle, the edge angle determines the skier’s ability to maintain grip, enter and exit turns, and control speed during the carving process. It is a key indicator for skiers to perform precise and efficient carving. By coordinating the knee and hip joint angles, skiers can manipulate the edge angle to ensure effective grip and overall control [32,33]. This study was approved by the Institutional Review Board of the Shanghai University of Sport (No. 102772021RT083) and was conducted in accordance with the guidelines of the Declaration of Helsinki. All procedures and potential hazards were clarified to the participants in nontechnical terms, and an informed consent was signed prior to the tests.

To validate the dynamic accuracy of SnowMotion, we focused on several key indicators that capture the essential aspects of skiing technique. These indicators include the left and right knee flexion angles ($\alpha_{L}$ and $\alpha_{R}$), which represent the angle between the thigh and the shank when the knee is bent. Additionally, we examined the left and right hip flexion angles ($\beta_{L}$ and $\beta_{R}$), which describe the angle between the trunk and the thigh when the hip is bent. Another crucial indicator is the edge angle, which is defined as the angle between the plane of the local snow surface and the running surface of the ski [34]. We considered both the left and right edge angles ($\gamma_{L}$ and $\gamma_{R}$) in our analysis. Lastly, we included the wedge angle (ω), which represents the horizontal angle between the two skis [35]. These indicators collectively provide a comprehensive assessment of the dynamic accuracy of SnowMotion in capturing and analyzing skiing technique. 

Based on previous studies, the sample size for the static validation was determined to be 26 repetitions of the action [36]. The right part of Figure 4 depicts the experimental setup, illustrating the positioning of optical markers and IMU sensor nodes on the body’s surface and the virtual model. The experiment tested three typical skiing movements: straight gliding, gliding wedge, and carving. The Vicon motion capture system was used to track and capture the spatial positions of each marker point and reconstruct the corresponding kinematic parameters using Visual3D software (v. 3.21.0), serving as the ground truth reference for comparison with the SnowMotion system output. The results are shown in Table 2.

## 4. Discussion

Through comprehensive testing and validation of the system, SnowMotion demonstrated exceptional motion capture accuracy and reliability, opening up new paths for intelligent assisted training in skiing. The experimental results indicate that the SnowMotion system can stably and precisely collect skiers’ kinematic data, with a motion capture accuracy cc of >0.95 against Vicon; a mean error of 5.033; an RMSE of <12.50 for typical movements like straight gliding, gliding wedge, and carving; a 60 Hz sampling rate; and <10 ms latency for real-time analysis. Through comprehensive testing and validation, SnowMotion has shown exceptional motion capture accuracy, reliability, and outstanding performance in real skiing scenarios, opening new paths for intelligent assisted training. These highlight SnowMotion’s potential to stably and precisely collect skiers’ kinematic data, aiding athletes and coaches.

In comparable skiing motion capture tasks, some studies have evaluated the performance of different functional calibration methods using inertial measurement units (IMUs) to record lower limb joint angles during gait in young, healthy individuals. These methods achieved an RMSE not exceeding 8.0 and a mean not exceeding 5.0 in terms of accuracy and repeatability [37]. In research focusing on center of mass (CoM) position estimation, a system demonstrated an accuracy and precision of less than 8.6 mm and 11.2 mm, respectively [38]. Another study analyzing the performance of IMU sensors in alpine skiing reported correlation coefficients between 0.66 and 0.75 when comparing the prototype system’s output with a reference system [39]. Considering that SnowMotion employs a minimized sensor count and a lightweight wearable design, the proposed system’s performance achieves state-of-the-art accuracy, demonstrating its effectiveness in capturing skiing motion while prioritizing user comfort and ease of use.

SnowMotion’s software interface uses a high-contrast color scheme and streamlined interactions for easy operation while wearing gloves. The hardware sensors were rigorously tested for low power consumption and waterproofing to adapt them to these conditions. To evaluate the system’s performance, we designed and conducted a series of ablation experiments to assess the impact of key modules, such as the sensor placement strategy, data fusion algorithms, and kinematic models for upper body motion inference. Optimal sensor placement on the lower limbs and torso significantly improved the motion capture accuracy. The proposed adaptive data fusion algorithm outperformed traditional approaches in terms of motion smoothness and stability. Incorporating biomechanical constraints and leveraging the body segment relationships in kinematic models substantially enhanced the accuracy of inferred upper body motion. These findings could guide further optimization, including refining the sensor placement, enhancing data fusion, improving the kinematic models, and exploring machine learning techniques for better performance and adaptability. Future work will explore co-designing the algorithm and sensor arrangement for joint optimization. Although no direct dependence exists currently, systematically evaluating their interplay could yield a holistic approach leveraging synergies, enabling efficient data processing, improved accuracy, robust systems, and practical real-world solutions. SnowMotion may spark a digital transformation in skiing by collecting vast amounts of data for big data analysis as the platform is widely adopted.

## 5. Conclusions

Looking ahead, the integration of technologies like IoT and big data provides immense optimization potential for SnowMotion. Intelligent algorithms could enable automatic scoring of ski movements and early warnings of injury risks, creating a true “personal digital coach.” In essence, SnowMotion represents an innovative fusion of cutting-edge technology and competitive sports training, showcasing the immense potential of tech-empowered sports. Future expansions to other sports and continuous iterative improvements could contribute significantly to the development of sports excellence.

## Figures and Tables

**Table 1:** RMSE and cc values from the forced pendulum experiment.

Frequency	RMSE	Mean	SD	cc	95% Confidence Interval
0.5 Hz	10.09	7.913	6.266	0.9842	7.709~8.118
1 Hz	10.90	6.321	7.063	0.9736	5.846~6.796
2 Hz	11.35	6.144	8.104	0.9743	4.858~5.619

RMSE: root-mean-square error; SD: standard deviation; cc: correlation coefficient.

**Table 2:** Key indicators for verification of dynamic accuracy.

Angle	RMSE	Mean	SD	cc	95% Confidence Interval
Straight Gliding					
$\alpha_{L}$	11.13	6.189	7.437	0.9607	5.514~6.483
$\alpha_{R}$	12.18	4.968	7.864	0.9756	4.247~5.055
$\beta_{L}$	12.36	5.527	6.744	0.9639	5.422~6.314
$\beta_{R}$	11.57	7.818	9.523	0.9618	7.305~8.792
$\gamma_{L}$	9.41	7.458	7.315	0.9843	7.775~8.011
$\gamma_{R}$	9.66	8.480	4.673	0.9671	8.798~9.835
ω	10.07	5.033	6.8	0.959	4.635~6.017
Gliding Wedge					
$\alpha_{L}$	12.04	5.887	9.533	0.9741	6.407~7.754
$\alpha_{R}$	11.27	5.305	8.091	0.9526	5.109~6.264
$\beta_{L}$	10.83	6.046	5.714	0.9551	6.244~6.889
$\beta_{R}$	10.5	7.977	8.906	0.9876	8.255~9.439
$\gamma_{L}$	9.47	6.002	7.238	0.9565	5.819~6.786
$\gamma_{R}$	11.93	6.914	6.188	0.9747	7.262~8.971
ω	8.69	7.203	4.207	0.9857	6.846~7.923
Carving					
$\alpha_{L}$	9.18	5.886	6.954	0.9598	5.183~6.854
$\alpha_{R}$	10.09	6.826	6.972	0.9738	6.641~7.229
$\beta_{L}$	11.05	6.901	7.804	0.9735	6.386~7.562
$\beta_{R}$	11.45	6.318	10.486	0.9537	5.865~7.188
$\gamma_{L}$	13.13	6.764	8.617	0.9807	6.301~7.449
$\gamma_{R}$	12.44	7.131	9.299	0.9577	5.818~7.773
ω	8.38	8.006	6.803	0.9972	7.325~8.451

RMSE: root-mean-square error; SD: standard deviation; cc: correlation coefficient.

## Data Availability

Dataset available on request from the authors.
